# From numbers to medical knowledge: harnessing combinatorial data patterns to predict COVID-19 resource needs and distinguish patient subsets

**DOI:** 10.3389/fmed.2023.1240426

**Published:** 2023-11-08

**Authors:** Parthkumar H. Satashia, Pablo Moreno Franco, Ariel L. Rivas, Shahin Isha, Abby Hanson, Sai Abhishek Narra, Kawaljeet Singh, Anna Jenkins, Anirban Bhattacharyya, Pramod Guru, Sanjay Chaudhary, Sean Kiley, Anna Shapiro, Archer Martin, Mathew Thomas, Basar Sareyyupoglu, Claudia R. Libertin, Devang K. Sanghavi

**Affiliations:** ^1^Department of Critical Care Medicine, Mayo Clinic, Jacksonville, FL, United States; ^2^Center for Global Health-Department of Internal Medicine, School of Medicine, University of New Mexico, Albuquerque, NM, United States; ^3^Mayo Clinic Alix School of Medicine, Jacksonville, FL, United States; ^4^Division of Cardiovascular and Thoracic Anesthesiology, Department of Anesthesiology and Perioperative Medicine, Mayo Clinic, Jacksonville, FL, United States; ^5^Department of Cardiothoracic Surgery, Mayo Clinic, Jacksonville, FL, United States; ^6^Division of Infectious Diseases, Mayo Clinic, Jacksonville, FL, United States

**Keywords:** personalized and prognostic medicine, resource allocation, early patient partitioning, structured blood leukocyte data, combinatorial immunology

## Abstract

**Background:**

The COVID-19 pandemic intensified the use of scarce resources, including extracorporeal membrane oxygenation (ECMO) and mechanical ventilation (MV). The combinatorial features of the immune system may be considered to estimate such needs and facilitate continuous open-ended knowledge discovery.

**Materials and methods:**

Computer-generated distinct data patterns derived from 283 white blood cell counts collected within five days after hospitalization from 97 COVID-19 patients were used to predict patient’s use of hospital resources.

**Results:**

Alone, data on separate cell types—such as neutrophils—did not identify patients that required MV/ECMO. However, when structured as multicellular indicators, distinct data patterns displayed by such markers separated patients later needing or not needing MV/ECMO. Patients that eventually required MV/ECMO also revealed increased percentages of neutrophils and decreased percentages of lymphocytes on admission.

**Discussion/conclusion:**

Future use of limited hospital resources may be predicted when combinations of available blood leukocyte-related data are analyzed. New methods could also identify, upon admission, a subset of COVID-19 patients that reveal inflammation. Presented by individuals not previously exposed to MV/ECMO, this inflammation differs from the well-described inflammation induced after exposure to such resources. If shown to be reproducible in other clinical syndromes and populations, it is suggested that the analysis of immunological combinations may inform more and/or uncover novel information even in the absence of pre-established questions.

## 1. Introduction

Medical decision-making is a vast and dynamic field that needs renewal and expansion. Hoping to improve its tools, here we explore the information potential of combinatorial immunology in the context of *patient stratification*, *allocation of limited resources*, and knowledge discovery related to *personalized medicine*.

This inquiry is grounded on the quasi-infinite combinatorial ability of the immune system, which allows it to do *more, faster or better, with less* (1). Immunology may resemble human language: while individual letters, alone, lack meaning, when combined, they become words that possess meaning. Similarly, individual cell types (e.g., neutrophils) may lack meaning. However, when combined with or integrated into multicellular groups they acquire meaning, i.e., the functions they perform may be deduced. While less than 30 letters can generate half a million words—as any dictionary can demonstrate—just five immunological cell types (neutrophils, monocytes, lymphocytes, basophils, and eosinophils), when combined, can protect from thousands of pathogens.

Combinatorial analysis may potentially distinguish patient groups that differ both in immunological profiles and outcomes based on recognition of data patterns (1). When pattern recognition emanates from interactions involving at least three dimensions, the relevance of any one input variable may be minor compared to the impact of all interactions—which, very frequently, are not known. Because the number of possible interactions rapidly grows when dimensions are added (including time), it may be impossible to predict what any one set of variables may induce. However, when biological knowledge is combined with a diverse set of validation studies, it may be possible to identify some data patterns that may have clinical implications.

To that end, validation studies should, at least, assess (i) whether the observed data patterns express or relate to features of biomedical relevance (construct validity), (ii) whether findings are not due to spurious or accidental relationships, which may disappear when different variables are analyzed (internal validity), (iii) whether findings are robust to differences in populations, geographical locations, time points and/or outcomes (external validity), and (iv) after the previous issues are addressed, whether differences among patient groups reach statistical significance (statistical validity).

Studies conducted over two decades have demonstrated that immunological data patterns can distinguish no inflammation from early and late inflammation (2). Some of such patterns are robust to differences in the (avian vs. mammalian and human vs. non-human) species investigated (3, 4). They may also apply to different diseases or syndromes, such as those induced by human immunodeficiency virus, hantavirus, COVID-19 and sepsis, and may be observed in populations located in several continents (5–8).

The properties of infectious disease-related data may have clinical applications. For example, the discovery of the inherent circularity of temporal observations of immunological profiles (which have no beginning and no end but cyclic or oscillatory patterns) means that numbers, alone, cannot distinguish biologically different phases (such as early from late inflammations). Yet, temporal data directionality (arrows that indicate the directionality of two consecutive observations) can differentiate such phases (3). A variation of the same concept is when complex, longitudinal and three-dimensional (3D) data structures reveal a single line of one data point-wide observations—a structure that eliminates data variability across all dimensions but one—the one characterized by a single line of points. Such structures, when arrows that indicate directionality are used, reveal, in real time, whether the data of a single patient moves toward the desirable or undesirable poles of the data and, consequently, facilitate earlier medical decision-making (7, 9). Real-time and personalized medical decision-making can also involve the evaluation of antibiotics: patterns of bacterial-immunological-antibiotic-temporal interactions can provide actionable information even before antibiogram test-results are available (10).

Given this background, here it is asked whether these informative capabilities may apply to manage and allocate critical hospital resources, such as mechanical ventilation (MV) and extracorporeal membrane oxygenation [ECMO (11, 12)]. Calls for ECMO-related, personalized decision-making have recently been made (13). They are motivated by the poor predictability of models that explore ECMO use in COVID-19 patients (14–16).

Distinguishing clinical varieties and stratifying patients on admission is another medical challenge (17). In addition to COVID-19, assessments of immune profiles may facilitate early patient partitioning and prognostics in other diseases, such as sepsis (18–20). Because such profiles are combinatorial—and, consequently, tens of thousands of immunological interactions may occur—a very large number of different research questions can be entertained even with the same data (21). The combinatorial analysis of immune profiles may also apply to personalized medicine (1, 20).

Pursuing deep learning—i.e., uncovering information usually hidden—*combinatorial experimentation* is closely related to *continuous knowledge discovery* and *causal reasoning* (22, 23). Because there is no consensus on its definition (23), combinatorial clinical research may be characterized as a partial re-analysis of the same continuous data, together with novel (previously untested) discrete variables or questions.

We followed a combinatorial approach to retrospectively analyze serial blood leukocyte (continuous) data to forecast the requirement for MV and ECMO in hospitalized COVID-19 patients. Based on evolutionary biology, it was hypothesized that immunological functions are unlikely performed by isolated units that act independently but could be performed by groups of two or more cell types (‘words’) that, through feedback processes conserved across host species, interact (3). The combinatorial expression of multicellular interactions may convey interpretable information (1, 24).

Because some multicellular interactions may perform different functions in different contexts and/or disease stages, a data-driven, combinatorial analysis may also result in unexpected knowledge discovery. Hence, this study aimed to determine whether data-driven research may offer intentional and unintentional knowledge discovery when a combinatorial analysis is conducted in clinical medicine. To that end, we investigated data partially explored elsewhere (9, 10) together with new information on COVID-19 patients.

## 2. Materials and methods

This study retrospectively collected serial complete blood cell counts (CBCs), and C-reactive Protein (CRP) data from 97 hospitalized COVID-19 patients admitted on or before August 2020 and evaluated them longitudinally, resulting in 283 temporal observations (Supplementary Table S1). While other molecules of potential diagnostic value were also evaluated (such as ferritin and lactate dehidrogenase), they are not reported here because they showed profiles similar to those of CRP.

According to internal institutional research board (IRB) protocol ID 21–002778, this retrospective study closely examined laboratory-confirmed 97 COVID-19 patients admitted to Mayo Clinic Florida. Inclusion required that the patient be older than 18, have SARS CoV-2 positive test results conducted within 72 h of admission and radiographic changes consistent with COVID, and be deemed at risk of severe illness to be enrolled. Subjects were excluded if they had a history of or were treated for immunosuppression, malignancy, pregnancy; had been exposed to MV/ECMO; and/or had been hospitalized for 3 or more weeks in the previous six months. At the end of the hospitalization (60 days or less), the use of hospital resources, including MV and ECMO, was determined with a scale that resembled the one reported by the World Health Organization’s Clinical Progression Scale (25). Because this study did not enroll ambulatory cases, the original WHO nine-point scale for infected individuals was reduced to a five-point scale, namely: (a) room air (scale #3), (b) nasal cannula (scale #4), (c) oxygen mask (scale #5), (d) high-flow cannula (scale #6), and (e) MV/ECMO (scale #7). For example, patients that required continuous positive airway pressure (CPAP) or bilevel positive airway pressure (BiPAP) were assigned scale ‘6’ (non-invasive, high-flow ventilation). Supplementary Table S2 summarizes the clinical features of each group of patients.

This study retrospectively analyzed the relationship between hospital resources identified at the end of the hospitalization period and blood leukocyte data collected in the first 5 days. The blood leukocyte data were structured using a proprietary software package (US Patent #10,429,389 B2), which generated pattern-revealing indices derived from leukocyte counts and/or relative percentages (here identified with one- to three-letter descriptors expressed in italics, e.g., *ABC*). These indices do not have any known biomedical meaning—they are identifiers that distinguish data combinations used as input variables. Because the patterns observed are outputs (i.e., the result of interactions that include triplets of such indices), these indices are only temporary guides required by the method investigated but no observed pattern is due to any one input variable.

### 2.1. Statistical analysis

This study was set to determine the ability of combinatorial experimentation to (i) predict the use of hospital resources according to an abbreviated version of the WHO Clinical Progression Scale (25) and (ii) extract more or new information from the same data. To that end, two analytical procedures were compared, which utilized non-structured and structured data, respectively. The first approach focused on primary data on counts and relative percentages of blood leukocytes, analyzed in isolation. The second approach investigated the same data after structuring performed with the algorithm identified above. Given that more than 95% of the tested individuals yielded <5% eosinophils and/or basophils, such cell types were not analyzed.

Findings elicited by structured and non-structured data analyses were compared considering several biologically interpretable variables. Comparisons across data subsets used the Mann–Whitney test for the median conducted by a commercial package *Minitab 19* (Minitab Inc., State College, PA, United States). To determine robustness, findings generated on hospitalization day 1 were compared to all longitudinal data entries.

## 3. Results

**Figure 1:**
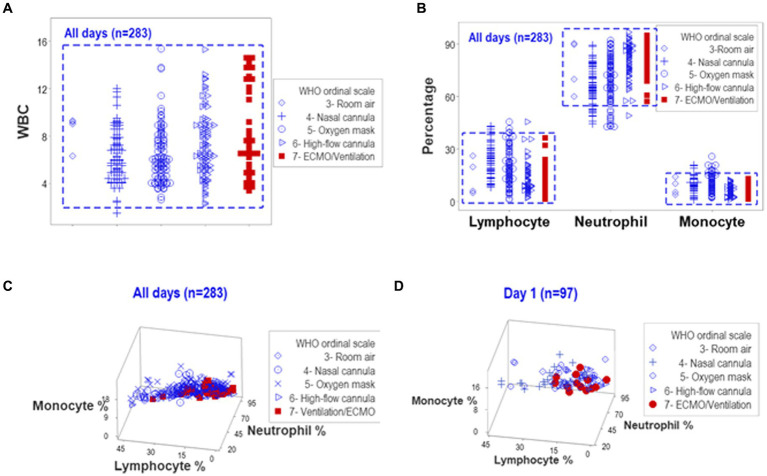
Analysis of unstructured data. Complete blood cell count (CBC) data collected from 101 COVID-19 patients over five days after hospitalization (283 temporal observations) were classified according to WHO ordinal scale. The uni-dimensional analysis of leukocyte counts (white blood cells or WBC) revealed overlapping data distributions that prevented the separation of observations according to the WHO scale [rectangle, **(A)**]. The analysis of different cell types, measured as relative percentages, also exhibited overlapping intervals across WHO classes [rectangle, **(B)**]. A three-dimensional analysis of the same data displayed overlapping intervals that prevented patient partitioning, both when all temporal observations were considered and when day-1 data were plotted **(C,D)**.

When all temporal observations collected from hospitalized COVID-19 patients were reviewed in an unstructured data format, overlapping counts and overlapping relative percentages of lymphocytes, neutrophils, and monocytes prevented differentiations among patients when the ordinal scale developed by WHO to distinguish COVID-19-related oxygenation treatments was used (Figures 1A,B). Hence, neither counts nor percentages distinguished patients who might need MV/ECMO from those who did not. Discrimination among treatments did not improve when three-dimensional relationships of the same data were assessed (Figures 1B,C). Data collected on hospitalization day (HD) 1 did not differentiate the WHO classes (Figure 1D).

**Figure 2:**
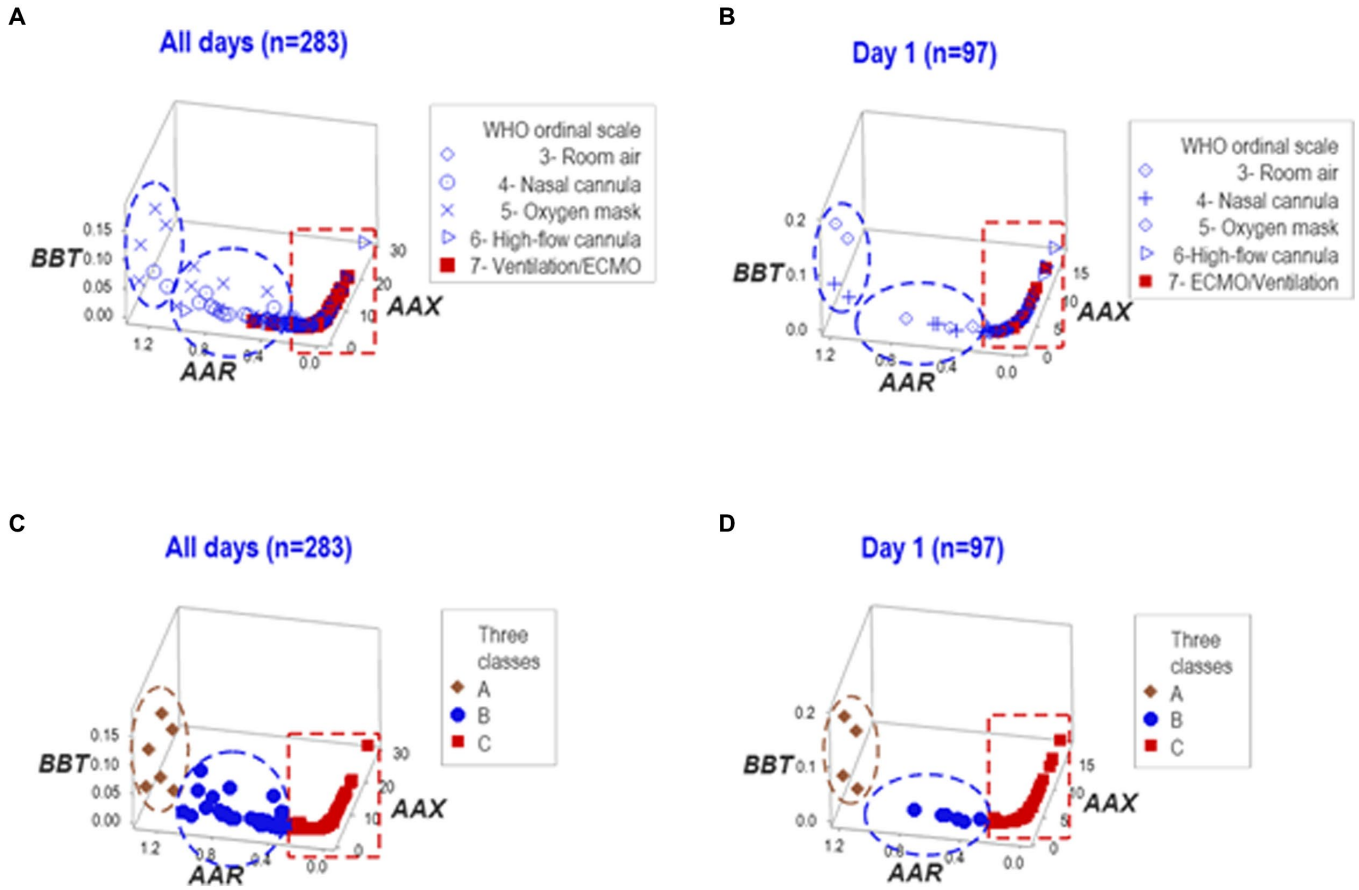
Analysis of structured data. The same data reported in Figure 1 were analyzed as structured data, i.e., hypothetical indicators that measure multicellular interactions and tend to reveal distinct patterns (here identified as two- or three-letter acronyms expressed in italics). Both the whole dataset and day-1 observations, when analyzed in three-dimensional space, expressed two data inflections that distinguished three groups of observations (‘A’,'B’, and ‘C’). ECMO/MV-related cases were clustered within the ‘C’ group **(A-D)**.

In contrast, when the same data were structured by a computerized algorithm, three distinct data patterns helped distinguish three non-overlapping data subsets in the longitudinal and HD 1 datasets (Figures 2A–D).

**Figure 3:**
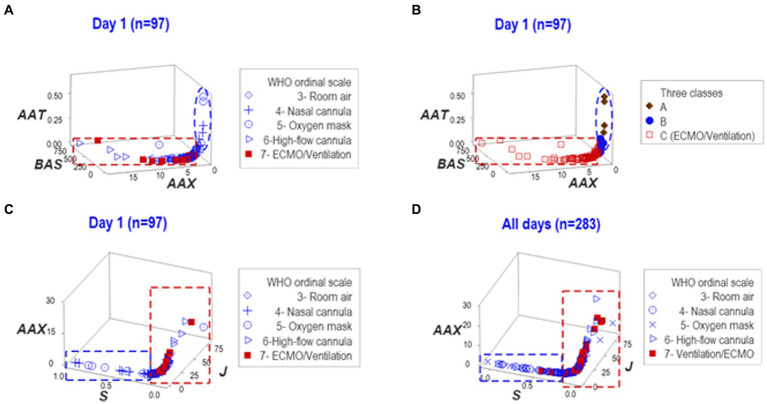
Reproducibility. To explore whether findings depended on a single data structure and/or temporal assessment, two additional sets of structured data were investigated—one at day 1, the other at both day 1 and all longitudinal observations. Such analyses distinguished the three **(A–C)** data classes detected in Figure 2 of which ECMO/MV clustered in one of them **(A,B)**. The third data structure exhibited two data subsets perpendicular to one another at day 1 and also when all temporal observations were considered **(C,D)**. In both assessments, all ECMO/MV-related observations were clustered in one subset (red rectangle, **C,D**). Therefore, findings demonstrated that inferences did not depend on any one data structure or temporal test.

To determine whether findings were dependent on any one data structure, the reproducibility of the findings was investigated with another data structure. Such analysis also considered the three (A-C) data subsets previously detected. A perpendicular data inflection revealed by the new analysis clustered patients requiring MV/ECMO into a separate group of observations (Figure 3A). Showing reproducible findings, the MV/ECMO group was again clustered within the ‘C’ class (Figure 3B).

When dynamics were explored with a third data structure, a perpendicular data inflection differentiated two groups of observations. In both assessments, one of such subsets did not include any ECMO/MV patient (Figures 3C,D).

**Figure 4:**
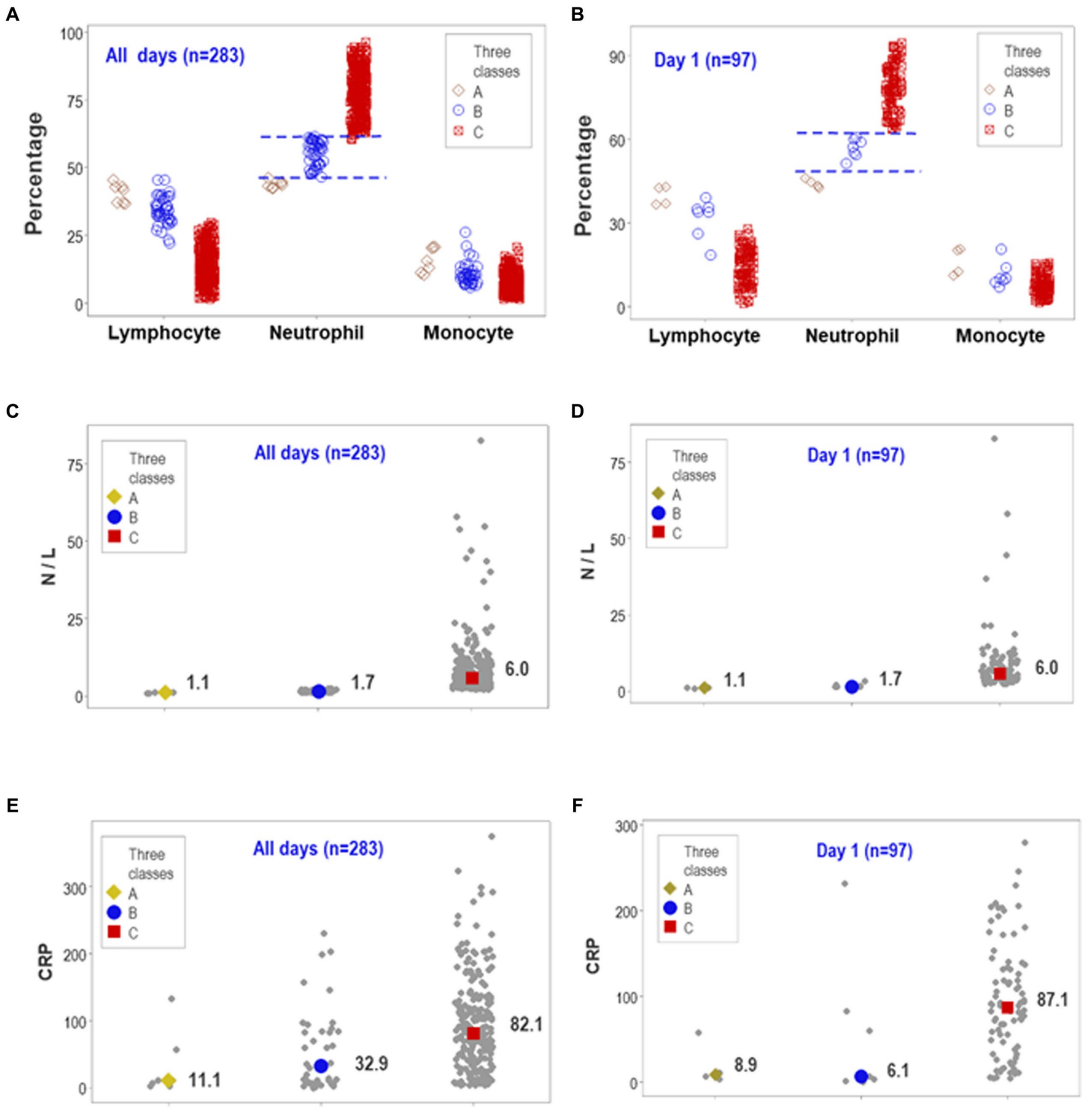
Predictability of personalized, biologically interpretable data and novel medical information. The ability of day-1 data to predict later use of MV/ECMO at personalized level was explored together with other considerations, including forecasting, patient stratification, personalized inferences and possible discovery. The similar patterns seen between the overall observations and day-1 data revealed both predictability and non-overlapping data intervals **(A,B)**. On day 1, the median neutrophil percentage displayed non-overlapping data intervals, differed by 15 or more percentage points among groups, and reached statistically significant differences between the ‘C’ and the remaining subsets (*p* < 0.01, Mann–Whitney test, B). The median neutrophil/lymphocyte (N/L) ratio also exhibited non-overlapping, statistically significant higher values between the ‘C’ and the remaining subsets **(C,D)**. In contrast, the concentration of C-reactive protein (CRP) revealed substantial data overlapping **(E,F)**. Therefore, full discrimination (when structured data revealed non-overlapping data subsets) could be associated with (but did not depend on) statistical significance. This predictable, biologically interpretable information also revealed a novel inference of medical value: because one subset of patients (those clustered in class ‘C’ or ECMO-related) presented with a proinflammatory profile not observed by other patient subsets, early (day-1) analysis of structured data facilitated the detection of or promoted (i) patient partitioning, (ii) personalized inferences (e.g., any patient classified within the ‘A’ or ‘B’ classes was not expected to require MV/ECMO), and (iii) a new inflammatory syndrome (presented by a subset of patients).

After documenting reproducibility, the predictability and biological validity were further investigated. The structured overall temporal and the structured day-1-only assessments displayed non-overlapping intervals of neutrophil percentages when the three data subgroups previously differentiated (‘A-C’ classes) were considered (Figures 4A,B). The median neutrophil percentage of the ‘C’ subset was significantly higher than the combined median of the ‘A’ and ‘B’ groups (*p* < 0.01, Mann–Whitney test). Therefore, data partitioning was immunologically and statistically valid.

Similarly, non-overlapping data distributions were found when the neutrophil/lymphocyte ratio (N/L) was considered (Figures 4C,D). The N/L medians also showed statistically significant differences between the ‘C’ subgroup (the one including MV/ECMO patients) and the remaining patients (*p* < 0.01, Mann–Whitney test, Figures 4C,D). In contrast, the CRP failed to display non-overlapping data intervals despite showing statistically significant differences at all-time points and when HD-1 data were analyzed (Figures 4E,F). Findings documented reproducibility and predictability: three different data structures and two temporal (day 1 and all longitudinal) assessments supported the view that non-overlapping discrimination and statistically significant differences could be achieved when structured data are analyzed.

## 4. Discussion

### 4.1. Overview

Three medical applications or novel findings associated with the immune profile of COVID-19 patients are discussed. One refers to *planning for using critical hospital resources*, one describes both *early patient stratification* and a *novel inflammatory pattern*, and one promotes *personalized medical decision-making* based on a *novel information system*.

### 4.2. Planning for the usage of critical hospital resources

At least two factors seem to influence specific patients’ estimated use of hospital resources: (i) lack of clinically interpretable information and (ii) analytical methods not fit for personalized medicine. While the planning literature on healthcare systems has considered both physical resources and the generation and allocation of human resources, biomedically explicit approaches (such as estimates based on patients’ immune profiles) have not yet been emphasized (26). While the need of personalized management has been articulated (13), it may be prevented by analyses that depend on relatively large sample sizes, i.e., data on population data where *n* > > 1. That is so because personalized clinical medicine involves *n* = 1 situations (8). Hence, assigning critical resources to specific patients requires personalized analysis of biomedically interpretable data.

To address such challenges, the advantages associated with prognostics and combinatorial immunology may be considered. The very high number of possible immunological *combinations* can be inferred from a simple observation: there are many more pathogens (~ 1,400) than the five cell types (lymphocytes, neutrophils, monocytes/macrophages, basophils, and eosinophils) that confer immunity (27). Such a few elements cannot act alone: only through combinations they can protect from infections, cancer and many other health disorders (1, 21). In addition, the immune system dynamics (even within the same patient) matter. Because even patients with the same diagnosis may experience different outcomes, diagnosis is not sufficient—*prognostics* are also needed, and they must be *individualized* (18).

These issues converge when the future usage of a critical resource is estimated. While the precise time when MV/ECMO would be required by a specific patient was not determined (because CBC data were only available for the first 5 HDs), individualized needs were estimated to materialize within 5 or more days after testing. If reproduced in other populations and clinical entities, this approach could address, simultaneously, several limitations of earlier methods—such as the errors associated with binary approaches (which do not apply when three or more alternatives exist), approaches that do not capture personalized physiologic inputs, *n* = 1 situations, and/or lack of biomedically interpretable information (28).

### 4.3. Early patient partitioning and inflammation as a predictor of MV/ECMO usage

While not pursued originally as a research goal, this study also demonstrated knowledge discovery (unintentional findings): a subset of patients was distinguished, since HD 1, by a proinflammatory immune profile. Such a finding differs from earlier studies on the same population, which focused on mortality (8). By focusing on the usage of MV/ECMO, new findings were uncovered: a subset of patients who, since HD1, presented with a non-overlapping, higher and statistically significant different interval of N% than two other groups of patients (Figures 4A,B).

This inflammation differs from the well-reported inflammation that may follow patient’s exposure to MV/ECMO (29, 30). The inflammation reported here may be detected since HD 1.

### 4.4. From assigning patients to pre-established diseases to dynamically monitoring disease evolution in personalized cases

This preliminary study supports the notion that combinatorial explorations of immunologic profile—if complemented with temporal, clinical, and personalized information—may both answer explicit research questions and offer (unintentionally) new usable information. Such a finding facilitates a new clinical/research practice in which continuous knowledge discovery is pursued and personalized medical decision-making is promoted.

While in population medicine the priority is to define data groups characterized by *n*> > 1 and then assign a specific patient to a previously defined disease class (an Oslerian or ‘one-size-fits-all approach), in personalized medicine (when *n* = 1) the individual is identified first. Because the patient may have specific co-morbidities and a previously unknown number of unique conditions, personalized characterizations precede other aspects and are considered when the diagnosis and prognosis are made, which should also evaluate, over time, whether the treatment modifies the course of the disease (31).

Hence, combinatorial assessments may induce not just one but at least four inferences, which refer to (i) the individual, (ii) the specific diagnosis, (iii) the future (prognostics), and (iv) possibly, many (non-binary) possibilities. That is potentially achieved when the dynamic and combinatorial properties of the immune system are considered and, instead of a single (one-time based) prediction, real-time, frequent assessments monitor disease progression at personalized levels (1, 32).

These considerations lead to propose tentative answers to a question previously asked: should the *case/control* model be replaced? (33). This question is motivated by several concepts ignored or erroneously assumed in the case/control approach, which include the fact that even patients that share the same diagnosis and receive the same treatment will differ over time and/or experience different outcomes (32). Far from being internally homogeneous, both ‘cases’ and ‘controls’ are heterogeneous and may differ in their dynamics (33).

These findings also highlight the *informative* relevance of *combinatorial immunology.* Given the quasi-infinite number of potential combinations (16, 21), even the same primary data may uncover new findings (8). More clinically useful information (that remains in data previously investigated) could be uncovered if *different questions* were asked, *more data patterns* were explored, and/or *new discrete* var*iables* were added.

Given the dysfunctional immune response observed in several diseases, such as COVID-19, it is suggested that this preliminary report may support additional combinatorial studies, which could account for personal heterogeneities and different dynamics. For example, different disease evolution in patients with or without immuno-deficiency could be explored (34). To that end, future population-based and personalized studies could explore combinations of cellular and non-cellular immunological markers in relation to specific treatments at specific times and/or in relation to specific co-morbidities and/or outcomes.

## 5. Caveats and conclusions

The expanded analysis of COVID-19 patients’ blood leukocyte data as structured indices may predict the need for using limited resources, such as ECMO and MV. This inquiry also revealed several novel findings or applications, including early patient stratification and the discovery of a pro-inflammatory profile in patients that later would be exposed to MV/ECMO. Provided that additional biologically meaningful variables are added and/or new questions are asked, it is suggested that combinatorial assessments may uncover more and/or new clinically useful information, even without new data.

## Data availability statement

The original contributions presented in the study are included in the article/Supplementary material, further inquiries can be directed to the corresponding author.

## Ethics statement

The studies involving humans were approved by Internal institutional research board (IRB) protocol ID 21-002778 of Mayo Clinic, Jacksonville, FL, USA. The studies were conducted in accordance with the local legislation and institutional requirements. The human samples used in this study were acquired from primarily isolated as part of your previous study for which ethical approval was obtained. Written informed consent for participation was not required from the participants or the participants' legal guardians/next of kin in accordance with the national legislation and institutional requirements.

## Author contributions

PS and PF: conceptualization, writing – original draft, writing – review & editing, visualization, supervision, and project administration. AR: conceptualization, writing – original draft, writing – review & editing, visualization, data curation, and data analysis. SI: original draft, writing – review & editing, visualization, supervision, and project administration. AH: conceptualization, writing – original draft, writing – review & editing, and manuscript preparation. SN, PG, SC, and KS: writing – original draft and writing – review & editing. AJ: writing – original draft and writing – review & editing. AB: writing – review & editing. SK, AS, AM, BS, and MT: writing – review & editing. CL and DS: conceptualization, writing – original draft, writing – review & editing, and visualization. All authors contributed to the article and approved the submitted version.
