# Supplementary material for: From numbers to medical knowledge: harnessing combinatorial data patterns to predict COVID-19 resource needs and distinguish patient subsets
Source: Front Med (Lausanne). 2023 Nov 8;10:1240426. doi: 10.3389/fmed.2023.1240426 (PMC10664024; doi:10.3389/fmed.2023.1240426)
Supplement: Supplementary file 1 [file Table_1.DOCX]

**Supplementary table 1. Immunological profiles of 97 COVID-19 positive patients tested in their first hospitalization days and their associated WHO scale (n=283)**

| Obs. | ID | Day | WHO scale | CRP | WBC | N | M | L | Three classes |
| --- | --- | --- | --- | --- | --- | --- | --- | --- | --- |
| 1 | 1 | 1 | 6- High-flow cannula | 202.80 | 8.1 | 81.4 | 7.0 | 11.5 | C |
| 2 | 1 | 2 | 6- High-flow cannula | 139.40 | 8.3 | 83.6 | 8.0 | 8.2 | C |
| 3 | 1 | 3 | 6- High-flow cannula | 74.20 | 11.4 | 86.5 | 5.8 | 7.5 | C |
| 4 | 1 | 4 | 6- High-flow cannula | 73.40 | 11.4 | 88.4 | 4.4 | 7.1 | C |
| 5 | 1 | 5 | 6- High-flow cannula | 7.60 | 12.9 | 91.4 | 3.2 | 5.2 | C |
| 6 | 2 | 1 | 6- High-flow cannula | 71.80 | 6.5 | 71.7 | 12.1 | 16.1 | C |
| 7 | 2 | 2 | 6- High-flow cannula | 156.80 | 6.3 | 57.4 | 6.9 | 35.5 | B |
| 8 | 2 | 3 | 6- High-flow cannula | 146.30 | 15.3 | 57.4 | 6.9 | 35.5 | B |
| 9 | 3 | 1 | 6- High-flow cannula | 153.40 | 13.2 | 93.3 | 4.1 | 2.5 | C |
| 10 | 3 | 2 | 6- High-flow cannula | 172.50 | 10.9 | 93.5 | 2.3 | 4.1 | C |
| 11 | 3 | 3 | 6- High-flow cannula | 73.40 | 12.1 | 88.4 | 6.0 | 5.4 | C |
| 12 | 4 | 1 | 6- High-flow cannula | 229.60 | 7.2 | 83.9 | 4.3 | 11.7 | C |
| 13 | 4 | 2 | 6- High-flow cannula | 119.20 | 6.0 | 83.4 | 4.0 | 12.5 | C |
| 14 | 4 | 3 | 6- High-flow cannula | 86.10 | 8.9 | 84.9 | 5.9 | 9.0 | C |
| 15 | 4 | 4 | 6- High-flow cannula | 83.10 | 8.9 | 78.5 | 6.4 | 14.9 | C |
| 16 | 5 | 1 | 6- High-flow cannula | 118.90 | 7.9 | 79.5 | 11.7 | 8.7 | C |
| 17 | 5 | 2 | 6- High-flow cannula | 118.60 | 6.8 | 76.4 | 11.7 | 11.7 | C |
| 18 | 5 | 3 | 6- High-flow cannula | 135.40 | 7.3 | 76.1 | 12.1 | 11.7 | C |
| 19 | 6 | 1 | 6- High-flow cannula | 108.20 | 9.0 | 88.0 | 4.8 | 7.0 | C |
| 20 | 6 | 2 | 6- High-flow cannula | 168.50 | 5.5 | 87.6 | 5.8 | 6.4 | C |
| 21 | 6 | 3 | 6- High-flow cannula | 109.90 | 6.3 | 82.7 | 8.5 | 8.7 | C |
| 22 | 6 | 4 | 6- High-flow cannula | 8.30 | 6.3 | 69.1 | 11.1 | 19.6 | C |
| 23 | 7 | 1 | 5- Oxygen mask | 9.90 | 5.4 | 65.9 | 15.4 | 18.6 | C |
| 24 | 7 | 2 | 5- Oxygen mask | 9.90 | 4.6 | 69.5 | 15.6 | 14.8 | C |
| 25 | 7 | 3 | 5- Oxygen mask | 6.20 | 2.6 | 52.3 | 25.8 | 21.8 | B |
| 26 | 8 | 1 | 5- Oxygen mask | 83.00 | 4.5 | 57.2 | 8.7 | 33.9 | B |
| 27 | 8 | 2 | 5- Oxygen mask | 122.50 | 9.1 | 79.6 | 8.5 | 11.8 | C |
| 28 | 8 | 3 | 5- Oxygen mask | * | 9.8 | 82.9 | 7.5 | 9.4 | C |
| 29 | 9 | 1 | 5- Oxygen mask | 4.50 | 7.7 | 70.4 | 12.7 | 16.8 | C |
| 30 | 9 | 2 | 5- Oxygen mask | 6.20 | 5.2 | 55.8 | 10.7 | 33.3 | B |
| 31 | 9 | 3 | 5- Oxygen mask | 10.00 | 6.3 | 58.2 | 13.5 | 28.1 | B |
| 32 | 10 | 1 | 5- Oxygen mask | 5.40 | 8.9 | 85.6 | 8.1 | 6.2 | C |
| 33 | 10 | 2 | 5- Oxygen mask | 5.40 | 7.7 | 92.2 | 3.0 | 4.7 | C |
| 34 | 10 | 3 | 5- Oxygen mask | 7.40 | 8.1 | 87.7 | 8.5 | 3.7 | C |
| 35 | 10 | 4 | 5- Oxygen mask | * | 11.4 | 85.6 | 9.7 | 4.6 | C |
| 36 | 11 | 1 | 5- Oxygen mask | 11.70 | 6.7 | 78.0 | 10.3 | 11.6 | C |
| 37 | 11 | 2 | 5- Oxygen mask | 7.00 | 7.4 | 71.6 | 10.4 | 17.9 | C |
| 38 | 11 | 3 | 5- Oxygen mask | 7.00 | 7.1 | 71.7 | 8.4 | 19.7 | C |
| 39 | 12 | 1 | 5- Oxygen mask | 11.10 | 4.0 | 42.5 | 20.5 | 36.8 | A |
| 40 | 12 | 2 | 5- Oxygen mask | 11.10 | 4.0 | 42.5 | 20.5 | 36.8 | A |
| 41 | 12 | 3 | 5- Oxygen mask | 20.20 | 3.5 | 46.3 | 17.4 | 36. | B |
| 42 | 13 | 1 | 5- Oxygen mask | 133.60 | 7.6 | 69.5 | 13.0 | 17.3 | C |
| 43 | 13 | 2 | 5- Oxygen mask | 133.60 | 4.3 | 43.0 | 15.3 | 41.5 | A |
| 44 | 13 | 3 | 5- Oxygen mask | 82.20 | 6.0 | 47.4 | 13.2 | 39.3 | B |
| 45 | 14 | 1 | 4- Nasal cannula | 12.90 | 6.0 | 76.7 | 7.2 | 16.0 | C |
| 46 | 14 | 2 | 4- Nasal cannula | 242.00 | 5.0 | 71.0 | 8.6 | 20.3 | C |
| 47 | 14 | 3 | 4- Nasal cannula | 10.50 | 4.4 | 48.7 | 13.2 | 37.9 | B |
| 48 | 15 | 1 | 4- Nasal cannula | 203.00 | 4.4 | 63.4 | 15.1 | 21.3 | C |
| 49 | 15 | 2 | 4- Nasal cannula | 203.00 | 4.0 | 55.7 | 20.9 | 23.2 | B |
| 50 | 15 | 3 | 4- Nasal cannula | 203.00 | 5.1 | 60.4 | 19.2 | 20.3 | C |

| Obs. | ID | Day | WHO scale | CRP | WBC | N | M | L | Three classes |
| --- | --- | --- | --- | --- | --- | --- | --- | --- | --- |
| 51 | 16 | 1 | 6- High-flow cannula | 104.90 | 4.40 | 80.51 | 8.28 | 11.21 | C |
| 52 | 16 | 2 | 6- High-flow cannula | 174.50 | 8.90 | 82.90 | 2.82 | 14.29 | C |
| 53 | 16 | 3 | 6- High-flow cannula | 132.60 | 8.90 | 94.77 | 1.91 | 3.32 | C |
| 54 | 17 | 1 | 5- Oxygen mask | 10.60 | 3.60 | 70.32 | 7.06 | 22.62 | C |
| 55 | 17 | 2 | 5- Oxygen mask | 13.50 | 3.90 | 60.55 | 6.80 | 32.66 | B |
| 56 | 17 | 3 | 5- Oxygen mask | 43.00 | 4.10 | 67.34 | 7.02 | 25.64 | C |
| 57 | 18 | 1 | 7- ECMO/ventilation | 85.60 | 4.90 | 83.95 | 3.99 | 12.07 | C |
| 58 | 18 | 2 | 7- ECMO/ventilation | 106.80 | 5.60 | 80.16 | 6.21 | 13.63 | C |
| 59 | 18 | 3 | 7- ECMO/ventilation | 70.40 | 7.60 | 80.59 | 7.11 | 12.30 | C |
| 60 | 19 | 1 | 7- ECMO/ventilation | 92.30 | 11.10 | 84.71 | 6.40 | 8.88 | C |
| 61 | 19 | 2 | 7- ECMO/ventilation | 141.90 | 7.40 | 77.66 | 5.17 | 17.17 | C |
| 62 | 19 | 3 | 7- ECMO/ventilation | 289.00 | 8.40 | 82.06 | 4.80 | 13.14 | C |
| 63 | 19 | 4 | 7- ECMO/ventilation | 22.40 | 14.00 | 92.69 | 2.84 | 4.47 | C |
| 64 | 19 | 5 | 7- ECMO/ventilation | 35.90 | 13.80 | 89.47 | 2.69 | 7.84 | C |
| 65 | 20 | 1 | 7- ECMO/ventilation | * | 13.80 | 93.35 | 5.04 | 1.61 | C |
| 66 | 20 | 2 | 7- ECMO/ventilation | * | 12.10 | 93.46 | 4.83 | 1.71 | C |
| 67 | 20 | 3 | 7- ECMO/ventilation | 375.20 | 12.80 | 93.62 | 4.05 | 2.33 | C |
| 68 | 20 | 4 | 7- ECMO/ventilation | 169.30 | 13.00 | 94.85 | 3.13 | 2.02 | C |
| 69 | 21 | 1 | 7- ECMO/ventilation | 204.20 | 7.60 | 81.14 | 2.24 | 16.62 | C |
| 70 | 21 | 2 | 7- ECMO/ventilation | 124.50 | 11.70 | 91.45 | 4.07 | 4.48 | C |
| 71 | 22 | 1 | 6- High-flow cannula | 278.60 | 8.60 | 89.18 | 1.92 | 8.90 | C |
| 72 | 22 | 2 | 6- High-flow cannula | 324.40 | 7.70 | 88.48 | 3.54 | 7.98 | C |
| 73 | 22 | 3 | 6- High-flow cannula | 292.70 | 7.40 | 88.70 | 2.32 | 8.98 | C |
| 74 | 23 | 1 | 5- Oxygen mask | 81.20 | 13.70 | 77.70 | 7.70 | 14.60 | C |
| 75 | 23 | 2 | 5- Oxygen mask | 65.00 | 13.80 | 83.67 | 2.51 | 13.82 | C |
| 76 | 23 | 3 | 5- Oxygen mask | 54.60 | 15.40 | 82.51 | 4.66 | 12.84 | C |
| 77 | 24 | 1 | 3- Room air | 138.60 | 9.20 | 89.84 | 5.33 | 4.83 | C |
| 78 | 24 | 2 | 3- Room air | 164.20 | 9.30 | 90.33 | 3.93 | 5.74 | C |
| 79 | 24 | 3 | 3- Room air | 82.00 | 9.00 | 70.03 | 10.20 | 19.77 | C |
| 80 | 25 | 1 | 5- Oxygen mask | 4.00 | 8.20 | 66.32 | 12.56 | 21.11 | C |
| 81 | 25 | 2 | 5- Oxygen mask | 3.70 | 7.10 | 64.51 | 12.62 | 22.87 | C |
| 82 | 25 | 3 | 5- Oxygen mask | 7.40 | 9.00 | 70.03 | 10.20 | 19.77 | C |
| 83 | 26 | 1 | 5- Oxygen mask | 113.70 | 8.30 | 81.87 | 8.16 | 9.97 | C |
| 84 | 26 | 2 | 5- Oxygen mask | 110.20 | 5.60 | 64.02 | 14.33 | 21.65 | C |
| 85 | 26 | 3 | 5- Oxygen mask | 45.00 | 3.80 | 74.07 | 7.04 | 18.89 | C |
| 86 | 27 | 1 | 7- ECMO/ventilation | 245.30 | 14.40 | 88.56 | 1.42 | 10.02 | C |
| 87 | 27 | 2 | 7- ECMO/ventilation | 214.30 | 12.80 | 91.32 | 2.02 | 6.66 | C |
| 88 | 27 | 3 | 7- ECMO/ventilation | 206.80 | 14.60 | 88.70 | 3.03 | 8.27 | C |
| 89 | 28 | 1 | 6- High-flow cannula | 32.40 | 5.10 | 79.76 | 5.44 | 14.80 | C |
| 90 | 28 | 2 | 6- High-flow cannula | 35.70 | 4.30 | 48.93 | 5.53 | 45.55 | B |
| 91 | 29 | 1 | 5- Oxygen mask | 92.50 | 5.40 | 73.24 | 6.14 | 20.62 | C |
| 92 | 29 | 2 | 5- Oxygen mask | 102.70 | 6.20 | 74.72 | 4.23 | 21.05 | C |
| 93 | 29 | 3 | 5- Oxygen mask | 80.50 | 4.60 | 65.39 | 6.46 | 28.15 | C |
| 94 | 30 | 1 | 7- ECMO/ventilation | 90.90 | 9.20 | 76.79 | 9.89 | 13.32 | C |
| 95 | 30 | 2 | 7- ECMO/ventilation | 101.40 | 5.40 | 85.65 | 3.93 | 10.42 | C |
| 96 | 30 | 3 | 7- ECMO/ventilation | 68.90 | 6.50 | 79.74 | 9.42 | 10.84 | C |
| 97 | 31 | 1 | 7- ECMO/ventilation | 99.50 | 6.50 | 77.92 | 4.84 | 17.24 | C |
| 98 | 31 | 2 | 7- ECMO/ventilation | 96.80 | 3.40 | 56.97 | 6.72 | 36.31 | B |
| 99 | 31 | 3 | 7- ECMO/ventilation | 46.10 | 14.60 | 71.80 | 9.00 | 19.20 | C |
| 100 | 32 | 1 | 6- High-flow cannula | 173.10 | 10.50 | 94.64 | 3.24 | 2.13 | C |

**Table 1 cont’d.**

**Table 1 cont’d.**

| Obs. | ID | Day | WHO scale | CRP | WBC | N | M | L | Three classes |
| --- | --- | --- | --- | --- | --- | --- | --- | --- | --- |
| 101 | 32 | 2 | 6- High-flow cannula | 175.80 | 10.5 | 89.81 | 3.13 | 7.06 | C |
| 102 | 32 | 3 | 6- High-flow cannula | 199.40 | 8.5 | 85.20 | 2.72 | 12.08 | C |
| 103 | 33 | 1 | 6- High-flow cannula | 144.50 | 9.4 | 92.84 | 2.82 | 4.33 | C |
| 104 | 33 | 2 | 6- High-flow cannula | 141.30 | 5.9 | 89.93 | 2.72 | 7.35 | C |
| 105 | 33 | 3 | 6- High-flow cannula | 68.30 | 8.3 | 86.29 | 4.64 | 9.07 | C |
| 106 | 34 | 1 | 4- Nasal cannula | 118.40 | 5.6 | 77.77 | 4.93 | 17.30 | C |
| 107 | 34 | 2 | 4- Nasal cannula | 116.80 | 5.7 | 72.40 | 6.17 | 21.44 | C |
| 108 | 34 | 3 | 4- Nasal cannula | 127.20 | 4.1 | 65.72 | 9.63 | 24.65 | C |
| 109 | 35 | 1 | 4- Nasal cannula | 58.90 | 4.1 | 75.83 | 6.45 | 17.72 | C |
| 110 | 35 | 2 | 4- Nasal cannula | 56.50 | 6.0 | 66.13 | 8.12 | 25.75 | C |
| 111 | 35 | 3 | 4- Nasal cannula | 28.90 | 7.9 | 67.88 | 7.85 | 24.27 | C |
| 112 | 36 | 1 | 6- High-flow cannula | 171.10 | 5.7 | 86.25 | 4.65 | 9.10 | C |
| 113 | 36 | 2 | 6- High-flow cannula | 196.30 | 5.1 | 86.09 | 5.83 | 8.08 | C |
| 114 | 36 | 3 | 6- High-flow cannula | 137.40 | 4.7 | 83.60 | 7.55 | 8.85 | C |
| 115 | 37 | 1 | 7- ECMO/ventilation | 125.50 | 6.9 | 90.23 | 1.91 | 7.85 | C |
| 116 | 37 | 2 | 7- ECMO/ventilation | 186.30 | 7.3 | 85.92 | 3.62 | 10.46 | C |
| 117 | 37 | 3 | 7- ECMO/ventilation | 113.90 | 8.0 | 85.73 | 5.33 | 8.94 | C |
| 118 | 38 | 1 | 5- Oxygen mask | 103.10 | 9.5 | 84.82 | 8.34 | 6.83 | C |
| 119 | 38 | 2 | 5- Oxygen mask | 85.60 | 6.2 | 80.56 | 7.45 | 11.98 | C |
| 120 | 38 | 3 | 5- Oxygen mask | 50.30 | 7.0 | 78.33 | 9.17 | 12.50 | C |
| 121 | 39 | 1 | 5- Oxygen mask | 0.00 | 5.4 | 51.28 | 9.50 | 39.22 | B |
| 122 | 39 | 2 | 5- Oxygen mask | 3.20 | 5.1 | 44.33 | 10.11 | 45.56 | A |
| 123 | 39 | 3 | 5- Oxygen mask | 4.50 | 4.9 | 47.93 | 6.71 | 45.35 | B |
| 124 | 40 | 1 | 4- Nasal cannula | 6.60 | 1.5 | 44.62 | 12.68 | 42.70 | A |
| 125 | 40 | 2 | 4- Nasal cannula | 7.20 | 2.4 | 52.61 | 7.53 | 39.86 | B |
| 126 | 40 | 3 | 4- Nasal cannula | 5.80 | 2.5 | 51.01 | 9.01 | 39.98 | B |
| 127 | 41 | 1 | 4- Nasal cannula | * | 8.1 | 65.57 | 9.73 | 24.69 | C |
| 128 | 42 | 1 | 5- Oxygen mask | 26.40 | 3.7 | 75.25 | 5.63 | 19.11 | C |
| 129 | 42 | 2 | 5- Oxygen mask | 28.30 | 3.5 | 76.96 | 6.64 | 16.40 | C |
| 130 | 42 | 3 | 5- Oxygen mask | 30.10 | 2.8 | 60.54 | 6.43 | 33.03 | B |
| 131 | 43 | 1 | 5- Oxygen mask | 82.90 | 8.1 | 87.94 | 4.22 | 7.84 | C |
| 132 | 43 | 2 | 5- Oxygen mask | 70.20 | 5.1 | 70.82 | 5.94 | 23.24 | C |
| 133 | 43 | 3 | 5- Oxygen mask | 52.10 | 7.2 | 78.35 | 7.65 | 14.00 | C |
| 134 | 44 | 1 | 4- Nasal cannula | 11.10 | 5.5 | 66.09 | 11.10 | 22.81 | C |
| 135 | 44 | 2 | 4- Nasal cannula | 7.60 | 5.5 | 67.57 | 10.62 | 21.81 | C |
| 136 | 44 | 3 | 4- Nasal cannula | 5.80 | 7.9 | 63.76 | 11.07 | 25.17 | C |
| 137 | 45 | 1 | 5- Oxygen mask | 205.30 | 10.1 | 84.63 | 3.13 | 12.23 | C |
| 138 | 45 | 2 | 5- Oxygen mask | 215.90 | 4.0 | 64.54 | 12.45 | 23.01 | C |
| 139 | 45 | 3 | 5- Oxygen mask | 127.30 | 9.7 | 66.56 | 10.83 | 22.61 | C |
| 140 | 46 | 1 | 6- High-flow cannula | 88.70 | 8.9 | 81.87 | 10.79 | 7.33 | C |
| 141 | 46 | 2 | 6- High-flow cannula | 87.80 | 5.5 | 87.01 | 5.79 | 7.21 | C |
| 142 | 46 | 3 | 6- High-flow cannula | 27.20 | 6.3 | 87.13 | 6.59 | 6.28 | C |
| 143 | 47 | 1 | 6- High-flow cannula | 83.10 | 6.8 | 76.85 | 5.99 | 17.16 | C |
| 144 | 47 | 2 | 6- High-flow cannula | 106.10 | 5.1 | 63.91 | 10.69 | 25.40 | C |
| 145 | 47 | 3 | 6- High-flow cannula | 57.80 | 6.3 | 64.52 | 9.38 | 26.11 | C |
| 146 | 48 | 1 | 6- High-flow cannula | 180.00 | 7.1 | 75.33 | 6.82 | 17.85 | C |
| 147 | 48 | 2 | 6- High-flow cannula | 102.00 | 6.5 | 74.67 | 6.43 | 18.89 | C |
| 148 | 48 | 3 | 6- High-flow cannula | 69.90 | 6.5 | 65.66 | 5.66 | 28.69 | C |
| 149 | 49 | 1 | 4- Nasal cannula | 6.10 | 3.6 | 59.03 | 6.96 | 34.01 | B |
| 150 | 49 | 2 | 4- Nasal cannula | 6.10 | 11.7 | 67.40 | 7.50 | 25.10 | C |

**Table 1 cont’d**

| Obs. | ID | Day | WHO scale | CRP | WBC | N | M | L | Three classes |
| --- | --- | --- | --- | --- | --- | --- | --- | --- | --- |
| 151 | 50 | 1 | 6- High-flow cannula | 46.60 | 10.7 | 86.2 | 7.1 | 6.5 | C |
| 152 | 50 | 2 | 6- High-flow cannula | 88.60 | 6.2 | 71.3 | 10.9 | 17.6 | C |
| 153 | 50 | 3 | 6- High-flow cannula | 54.40 | 4.7 | 75.5 | 9.1 | 15.3 | C |
| 154 | 51 | 1 | 6- High-flow cannula | 28.60 | 2.3 | 79.5 | 3.9 | 16.5 | C |
| 155 | 51 | 2 | 6- High-flow cannula | 22.50 | 4.1 | 73.9 | 5.1 | 20.9 | C |
| 156 | 51 | 3 | 6- High-flow cannula | 22.80 | 3.6 | 86.9 | 4.7 | 8.3 | C |
| 157 | 52 | 1 | 6- High-flow cannula | 92.90 | 8.1 | 84.5 | 6.2 | 9.1 | C |
| 158 | 52 | 2 | 6- High-flow cannula | 42.50 | 5.9 | 81.7 | 6.9 | 11.2 | C |
| 159 | 52 | 3 | 6- High-flow cannula | 101.50 | 12.8 | 90.8 | 3.7 | 5.4 | C |
| 160 | 53 | 1 | 7- ECMO/ventilation | 208.00 | 4.9 | 91.3 | 2.4 | 6.1 | C |
| 161 | 53 | 2 | 7- ECMO/ventilation | 133.60 | 4.6 | 88.1 | 4.4 | 7.4 | C |
| 162 | 53 | 3 | 7- ECMO/ventilation | 91.70 | 4.0 | 90.2 | 3.5 | 6.2 | C |
| 163 | 54 | 1 | 6- High-flow cannula | 140.50 | 5.3 | 86.2 | 5.5 | 8.2 | C |
| 164 | 54 | 2 | 6- High-flow cannula | 174.10 | 3.2 | 75.7 | 7.1 | 17.1 | C |
| 165 | 54 | 3 | 6- High-flow cannula | 88.60 | 3.8 | 67.1 | 12.2 | 20.5 | C |
| 166 | 55 | 1 | 6- High-flow cannula | 83.30 | 5.3 | 74.1 | 5.5 | 20.3 | C |
| 167 | 55 | 2 | 6- High-flow cannula | 112.60 | 4.2 | 67.4 | 7.1 | 25.3 | C |
| 168 | 55 | 3 | 6- High-flow cannula | 27.60 | 9.5 | 76.0 | 6.6 | 17.2 | C |
| 169 | 56 | 1 | 4- Nasal cannula | 71.80 | 9.5 | 73.3 | 7.5 | 19.0 | C |
| 170 | 56 | 2 | 4- Nasal cannula | 59.30 | 9.3 | 65.2 | 7.3 | 27.3 | C |
| 171 | 57 | 1 | 4- Nasal cannula | 15.10 | 7.8 | 70.5 | 15.5 | 13.8 | C |
| 172 | 57 | 2 | 4- Nasal cannula | 58.10 | 4.9 | 67. | 14.4 | 18.1 | C |
| 123 | 57 | 3 | 4- Nasal cannula | 20.10 | 11.3 | 80.4 | 10.0 | 9.4 | C |
| 174 | 58 | 1 | 5- Oxygen mask | 80.20 | 3.6 | 68.3 | 8.4 | 23.2 | C |
| 175 | 58 | 2 | 5- Oxygen mask | 84.10 | 3.9 | 57.6 | 9.6 | 32.6 | B |
| 176 | 58 | 3 | 5- Oxygen mask | 69.70 | 4.0 | 56.7 | 11.0 | 32.1 | B |
| 177 | 59 | 1 | 4- Nasal cannula | 174.50 | 10.6 | 80.6 | 7.5 | 11.8 | C |
| 178 | 59 | 2 | 4- Nasal cannula | 130.60 | 7.2 | 71.1 | 11.3 | 17.5 | C |
| 179 | 59 | 3 | 4- Nasal cannula | 11.60 | 5.7 | 59.5 | 10.9 | 29.5 | B |
| 180 | 60 | 1 | 5- Oxygen mask | 73.80 | 6.0 | 66.1 | 14.1 | 19.6 | C |
| 181 | 60 | 2 | 5- Oxygen mask | 74.00 | 4.4 | 72.2 | 14.9 | 12.8 | C |
| 182 | 60 | 3 | 5- Oxygen mask | 82.10 | 5.8 | 64.9 | 16.5 | 18.4 | C |
| 183 | 61 | 1 | 5- Oxygen mask | 13.80 | 5.5 | 68.3 | 11.9 | 19.7 | C |
| 184 | 61 | 2 | 5- Oxygen mask | 16.10 | 5.8 | 65.4 | 11.9 | 22.6 | C |
| 185 | 61 | 3 | 5- Oxygen mask | 12.80 | 5.7 | 60.4 | 12.6 | 26.8 | B |
| 186 | 62 | 1 | 4- Nasal cannula | 60.70 | 4.9 | 64.0 | 12.5 | 23.4 | C |
| 187 | 62 | 2 | 4- Nasal cannula | 28.60 | 5.7 | 63.2 | 12.5 | 24.2 | C |
| 188 | 62 | 3 | 4- Nasal cannula | 18.50 | 8.8 | 74.2 | 9.3 | 16.4 | C |
| 189 | 64 | 1 | 5- Oxygen mask | 15.20 | 5.7 | 84.4 | 14.4 | 1.0 | C |
| 190 | 64 | 2 | 5- Oxygen mask | 9.50 | 6.7 | 83.8 | 14.6 | 1.5 | C |
| 191 | 64 | 3 | 5- Oxygen mask | 8.20 | 4.1 | 62.0 | 17.1 | 20.7 | C |
| 192 | 65 | 1 | 7- ECMO/ventilation | 67.60 | 4.0 | 80.8 | 5.8 | 13.3 | C |
| 193 | 65 | 2 | 7- ECMO/ventilation | 68.40 | 6.3 | 75.4 | 11.3 | 13.2 | C |
| 194 | 65 | 3 | 7- ECMO/ventilation | 34.80 | 5.3 | 75.0 | 13.1 | 11.9 | C |
| 195 | 66 | 1 | 5- Oxygen mask | 73.80 | 6.0 | 66.3 | 14.1 | 19.5 | C |
| 196 | 66 | 2 | 5- Oxygen mask | 74.00 | 4.4 | 72.2 | 14.9 | 12.8 | C |
| 197 | 66 | 3 | 5- Oxygen mask | 85.90 | 5.8 | 64.9 | 16.5 | 18.4 | C |
| 198 | 67 | 1 | 4- Nasal cannula | 116.10 | 6.8 | 77.3 | 9.4 | 13.1 | C |
| 199 | 67 | 2 | 4- Nasal cannula | 110.20 | 6.8 | 74.2 | 9.4 | 16.3 | C |
| 200 | 67 | 3 | 4- Nasal cannula | 105.60 | 7.1 | 71.3 | 9.7 | 18.9 | C |

**Table 1 cont’d**

| Obs. | ID | Day | WHO scale | CRP | WBC | N | M | L | Three classes |
| --- | --- | --- | --- | --- | --- | --- | --- | --- | --- |
| 201 | 68 | 1 | 5- Oxygen mask | 49.30 | 6.1 | 81.53 | 8.70 | 9.77 | C |
| 202 | 68 | 2 | 5- Oxygen mask | 105.40 | 6.1 | 77.28 | 9.13 | 13.59 | C |
| 203 | 68 | 3 | 5- Oxygen mask | 51.70 | 5.2 | 79.35 | 9.36 | 11.29 | C |
| 204 | 69 | 1 | 5- Oxygen mask | 199.80 | 7.4 | 77.86 | 10.10 | 12.04 | C |
| 205 | 69 | 2 | 5- Oxygen mask | 193.10 | 7.6 | 89.93 | 4.83 | 5.24 | C |
| 206 | 69 | 3 | 5- Oxygen mask | 126.70 | 8.0 | 79.86 | 10.43 | 9.71 | C |
| 207 | 70 | 1 | 5- Oxygen mask | 131.20 | 4.0 | 76.91 | 5.42 | 17.67 | C |
| 208 | 70 | 2 | 5- Oxygen mask | 133.30 | 5.1 | 64.99 | 7.34 | 27.67 | C |
| 209 | 70 | 3 | 5- Oxygen mask | 56.50 | 6.9 | 74.17 | 7.16 | 18.67 | C |
| 210 | 71 | 1 | 5- Oxygen mask | 90.60 | 5.3 | 72.88 | 4.94 | 22.18 | C |
| 211 | 71 | 2 | 5- Oxygen mask | 84.00 | 7.3 | 61.24 | 8.95 | 29.81 | B |
| 212 | 71 | 3 | 5- Oxygen mask | 62.20 | 8.6 | 58.51 | 10.19 | 31.29 | B |
| 213 | 72 | 1 | 4- Nasal cannula | 22.80 | 4.0 | 64.61 | 9.14 | 26.25 | C |
| 214 | 72 | 2 | 4- Nasal cannula | 14.50 | 4.4 | 63.03 | 10.17 | 26.80 | C |
| 215 | 72 | 3 | 4- Nasal cannula | 13.30 | 4.9 | 60.51 | 9.18 | 30.31 | B |
| 216 | 73 | 1 | 3- Room air | 95.10 | 8.7 | 78.00 | 6.42 | 15.58 | C |
| 217 | 73 | 2 | 5- Oxygen mask | 115.10 | 8.8 | 80.79 | 3.05 | 16.16 | C |
| 218 | 73 | 3 | 5- Oxygen mask | 54.90 | 10.3 | 73.97 | 11.36 | 14.67 | C |
| 219 | 74 | 1 | 4- Nasal cannula | 231.10 | 5.9 | 55.05 | 10.21 | 34.74 | B |
| 220 | 74 | 2 | 4- Nasal cannula | 199.20 | 6.3 | 52.80 | 8.34 | 38.86 | B |
| 221 | 74 | 3 | 4- Nasal cannula | 97.80 | 6.9 | 51.73 | 8.60 | 39.66 | B |
| 222 | 75 | 1 | 4- Nasal cannula | 21.00 | 3.6 | 64.79 | 7.24 | 27.97 | C |
| 223 | 75 | 2 | 4- Nasal cannula | 37.70 | 4.3 | 61.55 | 7.57 | 30.88 | B |
| 224 | 75 | 3 | 4- Nasal cannula | 44.10 | 4.0 | 62.78 | 7.65 | 29.58 | C |
| 225 | 76 | 1 | 4- Nasal cannula | 22.80 | 12.0 | 79.86 | 10.27 | 9.87 | C |
| 226 | 76 | 2 | 4- Nasal cannula | 24.70 | 7.2 | 79.10 | 7.44 | 13.47 | C |
| 227 | 76 | 3 | 4- Nasal cannula | 33.60 | 5.3 | 68.18 | 12.93 | 18.89 | C |
| 228 | 77 | 1 | 5- Oxygen mask | 41.00 | 4.6 | 74.70 | 7.23 | 18.07 | C |
| 229 | 78 | 1 | 4- Nasal cannula | 55.50 | 7.3 | 82.91 | 7.74 | 9.35 | C |
| 230 | 78 | 2 | 4- Nasal cannula | 53.60 | 8.6 | 79.42 | 10.14 | 10.44 | C |
| 231 | 78 | 3 | 4- Nasal cannula | 42.60 | 8.9 | 77.32 | 9.68 | 13.00 | C |
| 232 | 79 | 1 | 5- Oxygen mask | 136.30 | 6.1 | 76.73 | 9.55 | 13.72 | C |
| 233 | 79 | 2 | 5- Oxygen mask | 122.60 | 8.9 | 82.53 | 7.50 | 9.97 | C |
| 234 | 79 | 3 | 5- Oxygen mask | 85.80 | 8.4 | 77.77 | 8.66 | 13.57 | C |
| 235 | 80 | 1 | 4- Nasal cannula | 67.60 | 9.0 | 69.73 | 7.27 | 23.01 | C |
| 236 | 80 | 2 | 4- Nasal cannula | 69.10 | 6.9 | 62.07 | 8.82 | 29.11 | C |
| 237 | 80 | 3 | 4- Nasal cannula | 77.00 | 6.8 | 67.41 | 9.37 | 23.22 | C |
| 238 | 81 | 1 | 4- Nasal cannula | 3.30 | 9.1 | 46.01 | 11.10 | 42.89 | A |
| 239 | 81 | 2 | 4- Nasal cannula | 3.00 | 9.1 | 49.07 | 9.79 | 41.14 | B |
| 230 | 81 | 3 | 4- Nasal cannula | 3.00 | 9.2 | 50.43 | 9.74 | 39.83 | B |
| 241 | 82 | 1 | 6- High-flow cannula | 72.30 | 12.2 | 86.85 | 6.83 | 6.32 | C |
| 242 | 82 | 2 | 6- High-flow cannula | 69.90 | 9.4 | 84.90 | 8.81 | 6.28 | C |
| 243 | 82 | 3 | 6- High-flow cannula | 60.80 | 11.9 | 86.25 | 5.46 | 8.29 | C |
| 244 | 83 | 1 | 7- ECMO/ventilation | 89.40 | 6.2 | 70.67 | 7.54 | 21.79 | C |
| 245 | 83 | 2 | 7- ECMO/ventilation | 94.60 | 3.8 | 60.56 | 7.42 | 32.03 | B |
| 246 | 83 | 3 | 7- ECMO/ventilation | 41.10 | 4.2 | 69.39 | 6.55 | 24.07 | C |
| 247 | 84 | 1 | 6- High-flow cannula | 16.30 | 2.9 | 85.86 | 6.92 | 7.22 | C |
| 248 | 84 | 2 | 6- High-flow cannula | 9.90 | 3.9 | 85.76 | 8.28 | 5.96 | C |
| 249 | 84 | 3 | 6- High-flow cannula | 6.60 | 4.2 | 88.24 | 5.73 | 6.03 | C |
| 250 | 85 | 1 | 5- Oxygen mask | 188.10 | 6.0 | 90.53 | 2.01 | 7.45 | C |

**Table 1 cont’d**

| Obs. | ID | Day | WHO scale | CRP | WBC | N | M | L | Three classes |
| --- | --- | --- | --- | --- | --- | --- | --- | --- | --- |
| 251 | 85 | 2 | 5- Oxygen mask | 181.00 | 4.0 | 89.55 | 2.71 | 7.74 | C |
| 252 | 85 | 3 | 5- Oxygen mask | 42.30 | 5.3 | 81.53 | 6.76 | 11.71 | C |
| 253 | 87 | 1 | 4- Nasal cannula | 55.80 | 6.2 | 81.96 | 8.77 | 9.27 | C |
| 254 | 87 | 2 | 4- Nasal cannula | 70.00 | 5.5 | 83.48 | 2.90 | 13.63 | C |
| 255 | 87 | 3 | 4- Nasal cannula | 61.00 | 5.0 | 73.54 | 1.23 | 25.22 | C |
| 256 | 88 | 1 | 6- High-flow cannula | 193.10 | 7.9 | 93.46 | 2.21 | 4.33 | C |
| 257 | 88 | 2 | 6- High-flow cannula | 125.50 | 7.2 | 96.68 | 1.11 | 2.22 | C |
| 258 | 88 | 3 | 6- High-flow cannula | 31.00 | 2.2 | 93.11 | 2.74 | 4.15 | C |
| 259 | 89 | 1 | 7- ECMO/ventilation | 6.00 | 6.5 | 68.93 | 10.15 | 20.91 | C |
| 260 | 90 | 1 | 7- ECMO/ventilation | 116.10 | 6.5 | 75.78 | 12.82 | 11.40 | C |
| 261 | 90 | 2 | 7- ECMO/ventilation | 257.10 | 3.7 | 76.61 | 9.38 | 14.01 | C |
| 262 | 90 | 3 | 7- ECMO/ventilation | 300.10 | 3.7 | 69.78 | 9.03 | 21.20 | C |
| 263 | 91 | 1 | 4- Nasal cannula | 59.90 | 4.1 | 54.45 | 10.12 | 35.43 | B |
| 264 | 91 | 2 | 4- Nasal cannula | 13.70 | 4.7 | 58.43 | 9.50 | 32.07 | B |
| 265 | 91 | 3 | 4- Nasal cannula | 8.40 | 5.2 | 64.75 | 8.40 | 26.84 | C |
| 266 | 92 | 1 | 4- Nasal cannula | 3.40 | 4.4 | 60.90 | 20.47 | 18.63 | C |
| 267 | 93 | 1 | 6- High-flow cannula | 37.80 | 3.1 | 72.35 | 8.07 | 19.58 | C |
| 268 | 94 | 1 | 5- Oxygen mask | 57.60 | 4.3 | 43.43 | 20.09 | 36.48 | A |
| 269 | 94 | 2 | 5- Oxygen mask | 88.00 | 3.9 | 59.38 | 11.74 | 28.89 | B |
| 270 | 94 | 3 | 5- Oxygen mask | 60.00 | 4.0 | 49.19 | 17.98 | 32.83 | B |
| 271 | 95 | 1 | 3- Room air | 1.10 | 6.3 | 59.91 | 14.01 | 26.08 | B |
| 272 | 96 | 1 | 6- High-flow cannula | 131.50 | 4.3 | 78.39 | 9.58 | 12.03 | C |
| 273 | 96 | 2 | 6- High-flow cannula | 160.00 | 5.0 | 78.09 | 9.94 | 11.97 | C |
| 274 | 96 | 3 | 6- High-flow cannula | 158.00 | 5.4 | 78.05 | 9.55 | 12.40 | C |
| 275 | 100 | 1 | 4- Nasal cannula | 77.60 | 8.6 | 89.18 | 1.92 | 8.90 | C |
| 276 | 100 | 2 | 4- Nasal cannula | 102.30 | 7.7 | 88.48 | 3.54 | 7.98 | C |
| 277 | 100 | 3 | 4- Nasal cannula | 103.00 | 7.4 | 88.70 | 2.32 | 8.98 | C |
| 278 | 101 | 1 | 4- Nasal cannula | 32.20 | 6.5 | 66.60 | 7.54 | 25.87 | C |
| 279 | 101 | 2 | 4- Nasal cannula | 19.10 | 8.9 | 78.87 | 7.94 | 13.20 | C |
| 280 | 101 | 3 | 4- Nasal cannula | 12.40 | 5.9 | 78.49 | 8.26 | 13.25 | C |
| 281 | 103 | 1 | 6- High-flow cannula | 110.50 | 6.2 | 89.18 | 1.92 | 8.90 | C |
| 282 | 103 | 2 | 6- High-flow cannula | 83.10 | 5.5 | 88.52 | 3.52 | 7.96 | C |
| 283 | 103 | 3 | 6- High-flow cannula | 61.40 | 8.5 | 88.70 | 2.32 | 8.98 | C |

Obs.: observation #. ID: patient identifier. Day: testing day (where 1 is the first hospitalization day). WBC: whole blood cell count (10^9^/L). WHO: WHO COVID-19 related clinical progression scale (18). CRP: C-reactive protein (mg/L). N, M, and L refer to the relative percentage of neutrophils, monocytes, and lymphocytes, respectively. Three classes (A, B, C): immunological profiles classified by an algorithm, where ‘C’ is associated with future needs for MV/ECMO.
