# Supplementary material for: From numbers to medical knowledge: harnessing combinatorial data patterns to predict COVID-19 resource needs and distinguish patient subsets
Source: Front Med (Lausanne). 2023 Nov 8;10:1240426. doi: 10.3389/fmed.2023.1240426 (PMC10664024; doi:10.3389/fmed.2023.1240426)
Supplement: Supplementary file 2 [file Table_2.DOCX]

**Supplementary Table 2. Baseline demographics and general comorbidities**

| Variable name | Total (n=283) | Non-survivors (n=37) | Survivors (n=246) | *p*-value |
| --- | --- | --- | --- | --- |
| Age | 61.00 (50.00, 70.00) | 71.00 (64.00, 78.00) | 59.00 (50.00, 69.00) | **<0.001** |
| BMI* | 29.18 (25.50, 33.92) | 26.69 (25.30, 31.40) | 30.20 (25.82, 34.16) | **0.03** |
| Gender  Male  Female | 176 (62.19%)  107 (37.81%) | 19 (51.35%)  18 (48.65%) | 157 (63.72%)  89 (36.18%) | 0.32 |
| Ethnicity  White  Black  Asian  Hispanic  Other | 217 (76.68%)  24 (8.48%)  12 (4.24%)  26 (9.19%)  4 (1.41%) | 32 (86.49%)  0 (0.00%)  3 (8.11%)  2 (5.41%)  0 (0.00%) | 185 (75.20%)  24 (9.76%)  9 (3.66%)  24 (9.76%)  4 (1.63%) | 0.14 |
| Hypertension | 96 (33.92%) | 16 (43.24%) | 80 (32.52%) | 0.19 |
| Coronary artery disease | 44 (15.55%) | 10 (27.03%) | 34 (13.82%) | **0.04** |
| Congestive heart failure | 5 (1.77%) | 0 (0.00%) | 5 (2.03%) | 0.38 |
| Hyperlipidemia | 47 (16.61%) | 8 (21.62%) | 39 (15.85%) | 0.37 |
| Chronic Kidney disease | 15 (5.30%) | 6 (16.22%) | 9 (3.66%) | **0.001** |
| Diabetes mellitus | 58 (20.49%) | 13 (35.14%) | 45 (18.29%) | **0.02** |
| Obesity | 7 (2.47%) | 0 (0.00%) | 7 (2.85%) | 0.29 |

*: Body mass index

Numbers within parentheses indicate the number of patients investigated, lower and upper limits of confidence intervals, and/or relative percentages corresponding to each column.
